# P01-08 Implementing school-based physical activity - putting Policy into Practice

**DOI:** 10.1093/eurpub/ckac095.008

**Published:** 2022-08-29

**Authors:** Thomas Skovgaard

**Affiliations:** Active Living, SDU, Odense M, Denmark

**Keywords:** School-based physical activity, Implementation science, Policy research, Factors of Change

## Abstract

Close to ten years ago, at the beginning of December 2012, the Danish government, announced its intention to implement comprehensive reforms of the public school system. Six months later, in June 2013, a broad-based political alliance agreed on extensive alterations in the legislation guiding Danish public schools.

The discussions of the most recent major reform of Danish public schools, established a next to unequivocal consensus among the parties in the Danish Parliament, school employers and employees as well as voluntary sports organizations that children and young people should be more physically active during the school day. Thus, the updated Danish Education Act of 2013 made it compulsory for public schools to offer an average of 45 minutes of physical activity (PA) per school day.

It is, however, painfully clear that the subsequent implementation of the primary policy goal to incorporate a minimum of 45 minutes of school-based PA for all pupils per day has been far from complete. According to some of the more exhaustive monitoring reports, in 2019 only six out of 10 schools fulfilled the basic PA-criterion. This was at the same level as in 2016. In spite of much political goodwill at both national, regional and local levels, significant investments of time and other resources by school authorities, professional organizations, a number of private and public foundations, individual employees and work teams, school managers and boards the number of schools actually adhering to current legislative goals related to physical activity has not changed in any substantial way from the adoption of the updated legislative framework in mid-2013 till present time.

Based on this state of affairs the presentation explores the question: What factors have influenced the translation of an overall policy ambition, related to school-based physical activity, into practice? The presentation builds on the Integrated Implementation Model, as presented by Winter and Nilsen and Cairney's introduction to Policy implementation research, and covers phases two and three of a total of four into which a given political decision-making process is typically divided: 1) agenda-setting, 2) policy generation, 3) implementation, and 4) evaluation.

